# A measure of modifiable lifestyle factors shaping subjective cognitive reserve in the general population

**DOI:** 10.3389/fpsyg.2024.1440076

**Published:** 2024-12-11

**Authors:** Carmen Moret-Tatay, José María Tormos Muñoz, Alvaro Pascual-Leone

**Affiliations:** ^1^Faculty of Psychology, Catholic University of Valencia San Vicente Mártir, Valencia, Spain; ^2^Faculty of Medicine and Health Sciences, Catholic University of Valencia San Vicente Mártir, Valencia, Spain; ^3^Department of Neurology, Harvard Medical School, Boston, MA, United States; ^4^Linus Health Inc., Boston, MA, United States

**Keywords:** subjective cognitive reserve, subjective decline, resilience, aging, general population

## Abstract

**Objective:**

This study examined the psychometric properties of a newly developed scale for measuring subjective cognitive reserve (SCR) across multiple domains, including nutrition, physical condition, sleep, cognition, willingness to learn, socialization, general health, and life plan.

**Method:**

The relationship between SCR scores and other established measures of cognitive reserve and subjective cognitive decline was also explored. A sample of 402 healthy participants aged 18 to 79 years took part in the study.

**Results:**

The SCR scale demonstrated strong psychometric properties, including internal consistency and construct validity, supporting the theoretical model of perceived cognitive reserve. Convergent validity was confirmed through a positive correlation between SCR scores and resilience (BRCS) as well as with other cognitive reserve measures, indicating consistency in evaluating cognitive reserve across various instruments. Furthermore, discriminant validity was demonstrated by a significant negative correlation between SCR scores and subjective cognitive decline, suggesting that individuals with higher cognitive reserve experience lower levels of perceived cognitive decline. No significant relationship was found between SCR scores and chronological age, further supporting the construct validity of the scale by showing that cognitive reserve is influenced by dynamic factors beyond age.

**Conslusion:**

The findings highlight the potential of the SCR scale as a reliable and valid tool for assessing cognitive reserve and its protective role in cognitive health and well-being over time.

## Introduction

Cognitive reserve (CR) has become an increasingly interesting topic in our society, captivating the attention of researchers worldwide. Despite the long-standing presence of various definitions in the literature, consensus has not always been unified, with terms like “cognitive reserve,” “brain reserve,” “resilience,” and “resistance” being used interchangeably (Cabeza et al., 2018; Stern et al., 2020, 2023), there seems to be agreement on its protective mechanism against age-related cognitive decline. In this way, the Collaboratory on Research Definitions for Reserve and Resilience in Cognitive Aging and Dementia, funded by the National Institute on Aging, established consensus definitions and research guidelines over a 3-year period starting in 2019. This framework, resulting from annual workshops and input from experts, defines CR, Brain Maintenance (BM), and Brain Repair (BR), offering operational definitions to aid research design. More precisely, CR is defined as “*a property of the brain that allows for cognitive performance that is better than expected given the degree of life-course related brain changes and brain injury or disease*” (Stern et al., 2023). Nevertheless, CR is also described to act against neurodegenerative disorders, enhancing adaptability to neurological challenges, and optimizing brain function (Cattaneo et al., 2022; McQuail et al., 2021). Thus, if one were to be able to enhance or sustain CR across the lifespan that would hold promise for individuals seeking to attenuate the effects of cognitive aging and neurodegenerative conditions, ultimately promoting healthy brain aging and quality of life.

For most, CR is being presented as a subtype of resilience as a homeostatic mechanism under the stress-response paradigm (Pascual-Leone and Bartres-Faz, 2021). While genetic factors certainly contribute to cognitive reserve CR, recent research underscores the importance of modifiable factors, such as lifestyle decisions, in shaping this resilience (Farina et al., 2021; Krivanek et al., 2021; Song et al., 2022). Understanding and dealing with these factors is important because they offer chances to improve cognitive abilities and possibly to reduce the risk of clinical manifestations or disability caused by cognitive decline and diseases like Alzheimer’s. Studies and various reports over time have shown that lifestyle changes, such as regular exercise, staying socially engaged, managing weight and blood pressure, maintaining mental health, and keeping the brain active, can help prevent more than 30% of dementia cases (Livingston et al., 2017, 2020, 2024). Not surprisingly, most questionnaire on CR described use some of these variables, such as education level (Song et al., 2022), whereas contemporary perspectives entertain the notion that even in later stages of life, various lifestyle elements have the potential to dynamically influence CR (Stern, 2009). In this scenario, several variables have been debated, such as education and cognition, socialization, vital plan, nutrition, sleep, general health, and physical condition (Cattaneo et al., 2018, 2020).

Framing the overall model of cognitive reserve CR reveals its multifaceted nature and the diverse factors that contribute to it. Educational attainment emerges as a key protective factor against cognitive decline, with higher levels of education associated with greater cognitive resilience (Lövdén et al., 2020; Stern et al., 1999). Engagement in intellectually stimulating activities throughout life also plays a crucial role in reducing the risk of dementia (Valenzuela and Sachdev, 2006). Socialization, too, is highlighted as a significant variable for CR, with strong social networks and engagement linked to a lower risk of cognitive decline (Fratiglioni et al., 2004). A comprehensive approach to health and wellbeing, encompassing factors such as balanced nutrition, regular exercise, quality sleep, and effective stress management, is emphasized as vital for fostering CR (Cheng, 2016; Erickson et al., 2011; Irigaray et al., 2022). Additionally, consistent physical activity is shown to offer neuroprotective benefits, maintaining neuronal integrity, and enhancing cognitive function (Farina et al., 2021; Northey et al., 2018). Quality sleep is crucial for memory consolidation, learning, and brain plasticity, while nutrition, characterized by essential nutrients, supports cognitive function and physiological robustness (Casagrande et al., 2022; Cellini, 2017; Curcio et al., 2006; Yaremchuk, 2018). Overall, addressing these various factors holistically can significantly impact an individual’s overall sense of coherence and cognitive resilience, promoting healthy brain aging and quality of life.

Quantifying CR presents a significant challenge, leading to the utilization of diverse methodologies across current research studies (Kartschmit et al., 2019). Many researchers commonly use indirect indicators, such as subjective questionnaires, to examine cognitive reserve. While some focus solely on one proxy of CR (Chapko et al., 2018; Kartschmit et al., 2019), often education, it is crucial to acknowledge that relying solely on a single indicator may not fully capture the multifaceted nature of CR, given its complexity and reliance on diverse components. Therefore, this study considers the role of various modifiable lifestyles in CR. The study aims to examine the psychometric properties of a new scale designed to measure subjective cognitive reserve (SCR), and the relationship between SCR measured by this scale and other relevant variables in the field such as resilience and age. Our hypotheses were:

H1: The proposed subjective cognitive reserve measure (SCR) depicts good psychometric properties for the general population. This result would provide empirical support for the current model of perceived CR among individuals.

H2: There is a positive correlation between SCR scores and scores on other established cognitive reserve (CR) questionnaires, as well as resilience measures. This suggests that individuals who score higher on the SCR scale are likely to obtain similar results on alternative CR assessments, indicating the criterion validity (convergent) of the SCR in measuring cognitive reserve consistently across different instruments. Moreover, this correlation supports the idea that individuals with greater cognitive reserve also tend to have higher resilience.

H3: There is a negative correlation between SCR scores and subjective cognitive decline scores. This would demonstrate discriminant validity, suggesting that the SCR scale can effectively distinguish between individuals with varying levels of perceived cognitive decline, further supporting its ability to measure cognitive reserve as distinct from cognitive decline. In other words, depicting criterion and discriminant validity.

H4: There is no significant relationship between SCR scores and chronological age. This would provide evidence for the construct validity of the SCR measure, indicating that cognitive reserve is not solely determined by age and can be influenced by other dynamic factors throughout life.

## Methods

### Participants

A total of 402 participants volunteered for the study. They were randomly divided into two groups for both exploratory and confirmatory analyses. In this way, the sample is divided into two groups to ensure independence between exploratory and confirmatory factor analyses (EFA and CFA), a method recommended to avoid bias (Brown, 2015). EFA is used to explore underlying factor structures, while CFA is conducted on a separate sample to confirm the factor structure, reducing the risk of overfitting (Worthington and Whittaker, 2006). This approach improves the reliability and generalizability of the findings, ensuring the factor model holds up across different samples (Brown, 2015). There were 201 participants in each group, with 70.1% of the participants being women in both groups.

For the EFA, the average age within this subgroup was 34.05 (*SD* = 14.91) and a range age from 18 to 77 years. The distribution of educational levels: A total of 0.5% had no studies, ≥ 6 education years is accounted for constituting 1%, ≥ 9 education years reached a 16.9% of the sample, a 81.6%, have achieved higher education. A 22.4% were unskilled (including “homemakers”), 10% worked as skilled manual workers, 12.9% worked as skilled non-manual workers, 47.8% worked as professionals, and 7% worked as managers or supervisors. With regards to marital status, 26.9% were “Married or in a civil partnership,” 6.5% are “Divorced or separated,” 26.9% are “In a relationship,” 39.3% were “Single,” and 0.5% were “Widowed.”

For the CFA, the sample resembled the previous one in its composition. The average age of participants in this group was 32.97 years (SD = 14.98), ranging from 18 to 79 years old. Regarding educational levels among the 162 study participants, 1% reported having no formal education, while 0.5% had completed at least 6 years of education. Additionally, 26.4% had completed at least 9 years of education, and the majority, comprising 72.1% of the sample, had attained higher education qualifications. Regarding occupational status, 27.4% were categorized as unskilled workers, which includes homemakers, while 7.5% were engaged in skilled manual labor, and 8% were in skilled non-manual occupations. Furthermore, 49.8% of participants worked in professional roles, while 7.5% held managerial or supervisory positions. In terms of marital status, 33.3% were married or in a civil partnership, 3% were divorced or separated, 26.4% were in a relationship, 38.8% were single, and 1.5% were widowed.

### Procedure

The development of the SCR involved a systematic process that incorporated input from an expert group with expertise in neuroscience, public health, and related fields. Initially, researchers conceptualized the construct they aimed to assess based on existing literature and expert knowledge. Subsequently, the group participated in multiple rounds of iterative discussions to refine the conceptual framework and identify relevant domains and items for inclusion in the questionnaire. These items underwent further evaluation to ensure clarity, relevance, and comprehensiveness ensuring they achieved a scoring of over 0.8 on a scale from 0 to 1. Pilot testing with a small sample was conducted to assess item performance, and revisions were. Ethical clearance was obtained from the institution research committee (UCV/2020–2021/16).

### Materials

All participants answered a battery of sociodemographic questions, as well as different scales, described as follows:

*The cognitive reserve questionnaire (CRC):* It consists of eight items with between three and six response options in its Spanish proposal (Rami et al., 2011). Each item assesses a cognitive reserve factor: education, parental education, courses, occupation, musical training, languages, reading, and intellectual games. The maximum score is 25, and the higher the score, the greater the cognitive reserve.

*The subjective cognitive decline questionnaire* (*SCD-Q*) in its Spanish adaptation (Rami et al., 2014). This consists of 24 items and is divided into two parts, the participant’s section (Part I, also called MyCog) and their informants’ section (Part II, also called TheirCog). In this case, only the participant’s version (MyCog) was used. The items in the SCD-Q are designed to capture various dimensions of subjective cognitive decline, even though the original authors did not explicitly categorize them into distinct domains. These dimensions include memory function (e.g., difficulty remembering recent conversations or events), attention and concentration (e.g., challenges in maintaining focus during activities), language skills (e.g., problems finding the right words during conversations), and executive functioning (e.g., difficulties in planning and organizing tasks). Together, these aspects aim to offer a comprehensive assessment of an individual’s subjective cognitive experiences. The specific questions for these areas are available in the original manuscript by Rami et al. (2014).

*The brief resilient coping scale (BRCS)* in its Spanish adaptation (Moret-Tatay et al., 2015). This consists of 4 items and uses a Likert response format with 5 anchor points, from 1 (does not reflect me at all) to 5 (reflects my usual way). With a 95% confidence intervals, the frequentist reliability analysis for the scale yielded McDonald’s omega (*ω*) and Cronbach’s alpha (*α*) values of 0.706 (0.655, 0.750) and 0.705 (0.659, 0.753), respectively, indicating an acceptable level of internal consistency.

*The subjective cognitive reserve (SCR)* proposed in this study. As described in Appendix A of this study, the questionnaire comprises eight items that assess perceptions regarding one’s nutrition, physical condition, sleep quality, cognitive function, willingness to learn, social interaction, overall health, and Vital plan (see Appendix A). The frequentist reliability analysis for the whole data set through the scale yielded McDonald’s omega (ω) and Cronbach’s alpha (α) values of 0.758 and 0.756, respectively, indicating acceptable internal consistency. The 95% confidence intervals for McDonald’s omega ranged from 0.722 to 0.794, while those for Cronbach’s alpha ranged from 0.718 to 0.790.

### Design and analyses

The study employed a psychometric analysis paradigm aimed at assessing the reliability and validity of the newly developed Subjective Cognitive Reserve (SCR) scale. The analyses were carried out using IBM SPSS 21, JASP 0.14.1.0, and AMOS 21 software. The psychometric properties were evaluated by examining both construct validity and criterion validity. Specifically, exploratory factor analysis (EFA) was used to identify potential underlying factor structures, and confirmatory factor analysis (CFA) was employed to test the fit of the proposed model using a separate sample, thus ensuring robustness. The reliability was assessed before creating subgroups and reassessed after the EFA to ensure that the factors derived are consistent and reliable, but it is not a component of the factor analysis itself. The assessment of reliability included both Cronbach’s alpha and McDonald’s Omega, which provided insight into the internal consistency of the SCR scale. These measures of reliability are part of a broader framework, which also covers test–retest reliability, though the study primarily focused on internal consistency in this context. To evaluate convergent and divergent validity, correlations were computed between SCR scores and related variables such as the Cognitive Reserve Questionnaire (CRC), Brief Resilient Coping Scale (BRCS), and Subjective Cognitive Decline Questionnaire (SCD-Q). Model adequacy for the CFA was confirmed using absolute the following fit indices: chi-square statistic (*χ*^2^), along with other key indices such as the Comparative Fit Index (CFI), with a reference value of 0.90 (Bentler, 1990), and the Root Mean Square Error of Approximation (RMSEA), for which values below 0.05 indicate a good model fit (Rigdon, 1996). Pearson’s correlation to assess the linear relationships between variables in this study. Furthermore, linear regression analysis was conducted to explore the relative contribution of various predictor variables (e.g., CRC, BRCS) in explaining the variance in subjective cognitive decline scores. This approach aims to ensure a comprehensive examination of both the dimensionality of the SCR scale and its relationships with external constructs.

## Results

With regards to the EFA (*n* = 201), reliability was reassessed using McDonald’s *ω* and Cronbach’s *α* coefficients, although this is not an intrinsic step of EFA. Both coefficients stand at an adequate level (0.784 for ω and 0.777 for α) for the internal consistency in the scale. The 95% confidence intervals provide additional insights, with lower bounds of 0.739 (ω) and 0.727 (α), and upper bounds of 0.829 (ω) and 0.820 (α). These intervals suggest a reasonably precise estimation, indicating that the true reliability of the scale is likely to fall within these ranges. Table 1 depicts frequentist Individual Item reliability statistics, including information on item correlation and the impact on reliability if a specific item is dropped, among others.

**Table 1:** Frequentist individual item reliability statistics.

**Item**	**If item dropped**				
	**McDonald’s *ω***	**Cronbach’s *α***	**Item-rest correlation**	**Mean**	***SD***
Nutrition	0.741	0.738	0.575	3.697	0.867
Physical condition	0.764	0.762	0.485	2.925	1.300
Sleeping	0.786	0.779	0.342	3.269	1.038
Cognition	0.764	0.755	0.479	4.020	0.781
Willingness to learn	0.769	0.760	0.438	4.338	0.803
Socialization	0.766	0.756	0.463	4.075	0.866
General health	0.743	0.738	0.584	3.950	0.817
Vital plan	0.750	0.739	0.579	4.139	0.831

The KMO Measure, with a value of 0.835, indicated a high level of sampling adequacy, suggesting that the data is suitable for factor analysis. On the other hand, the Bartlett’s Test of Sphericity yielded an approximate chi-square value of 388.157 (df = 28) and *p <* 0.000, supporting the rejection of the null hypothesis that the correlation matrix is an identity matrix, and confirming the presence of significant relationships between variables and justifying the use of factor analysis.

**Figure 1:**
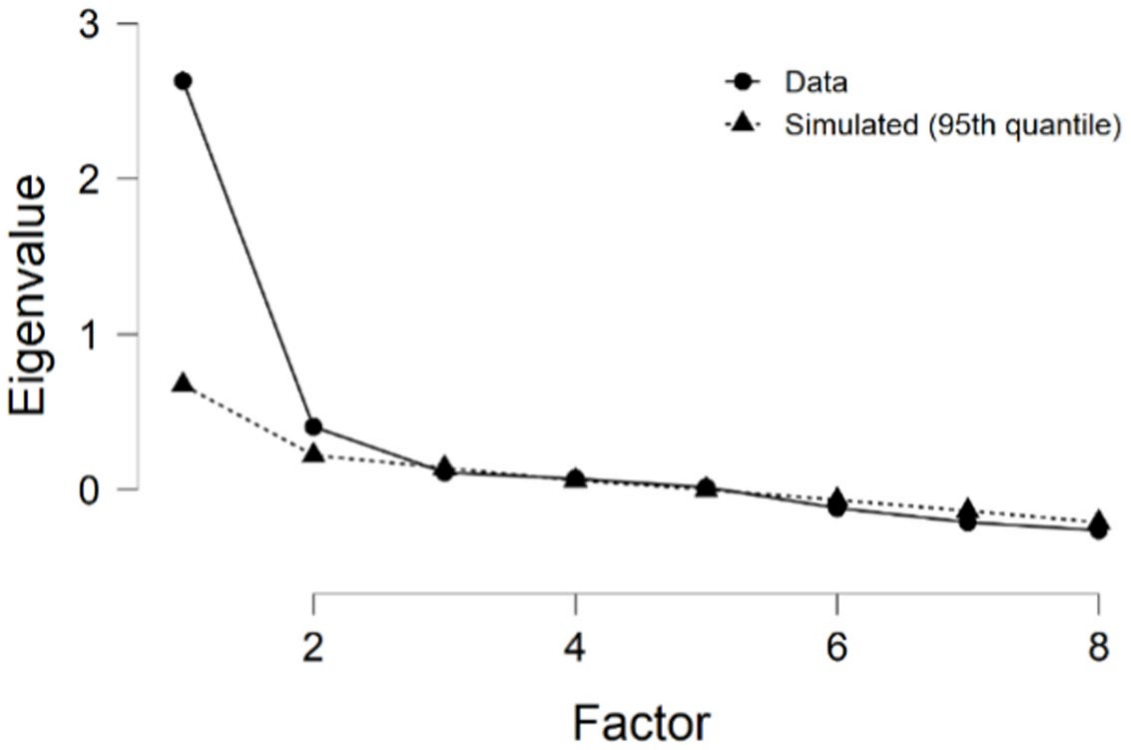
Scree plot in the parallel analysis.

The Cattell’s eigenvalue criterion revealed a factor with a 58% explained variance. Additionally, the parallel analysis results are illustrated in Figure 1. The factor loadings are displayed in Table 2, providing insights into the relationship between variables and the identified factor based on covariance, as well as uniqueness understood as the proportion of the variable’s variance that is not explained by other variables in the model. Although self-rating eigenvalue criterion and parallel analysis seem to indicate more than one factor, the factor loading appears to be concentrated on a single factor, suggesting a one-factor solution.

**Table 2:** Factor loadings in the EFA for 2 and 3 solutions.

	**One-factor solution**			**Two-factor solution**	
	**Factor 1**	**Uniqueness**	**Factor 1**	**Factor 2**	**Uniqueness**
Nutrition	0.585	0.585	0.681	−0.359	0.407
Physical condition	0.682	0.682	0.601	−0.394	0.483
Sleeping	0.845	0.845	0.385	0.036	0.850
Cognition	0.712	0.712	0.534	0.184	0.681
Willingness to learn	0.726	0.726	0.539	0.332	0.599
Socialization	0.702	0.702	0.545	0.210	0.659
General health	0.564	0.564	0.650	−0.111	0.565
Vital plan	0.553	0.553	0.669	0.221	0.504

No applied rotation method.

Secondly, a CFA (*n =* 201) was carried out in an independent subgroup from the sample. The Table 3 provides factor loadings along with their 95% confidence intervals for various indicators in a factor analysis, representing the strength and direction of the relationship between each indicator and the underlying factor. Table 3 indicates the factor loading for the CFA.

**Table 3:** Factor loadings in the CFA.

						95% confidence interval	
Factor	Indicator	Estimate	Std. error	*z*-value	*p*	Lower	Upper
Factor 1	Nutrition	0.565	0.060	9.357	< 0.001	0.446	0.683
	Physical condition	0.752	0.093	8.105	< 0.001	0.570	0.934
	Sleeping	0.410	0.078	5.264	< 0.001	0.258	0.563
	Cognition	0.407	0.057	7.179	< 0.001	0.296	0.519
	Willing to learn	0.417	0.058	7.131	< 0.001	0.302	0.531
	Socialization	0.468	0.063	7.488	< 0.001	0.346	0.591
	General health	0.542	0.057	9.588	< 0.001	0.431	0.653
	Vital plan	0.544	0.058	9.428	< 0.001	0.431	0.657

The results of the confirmatory factor analysis CFA indicate the goodness of fit for the model. The *χ*^2^ showed was statistically significant (*χ*^2^ = 69.642, df = 20, *p* < 0.001), as proposed in H_1_. Other fit indices should be considered. The comparative fit index (CFI), Tucker-Lewis index (TLI), Bentler-Bonett Non-normed Fit Index (NNFI), and Bentler-Bonett Normed Fit Index (NFI) all indicate moderate to good fit (CFI = 0.938, TLI = 0.908, NFI = 0.894). Additionally, the root mean square error of approximation (RMSEA) is 0.078 exceeded the commonly recommended threshold. Overall, the fit indices suggest a reasonably good fit.

After collecting initial data, a test–retest was conducted in the SCR after a period of 2–3 weeks to assess the reliability of the measurements. A subsample of 20 participants volunteered to participate again. The obtained result was 0.764, with a significance level of *p* < 0.01. This indicates a statistically significant correlation between the two sets of measurements. In other words, the data collected at the two different time points were highly consistent, suggesting good reliability of the measurement instrument or method used in the study. The high correlation coefficient implies that the measurements are stable over time.

Lastly, correlation and regression analyses were conducted using the full data set (*N* = 402). Table 4 presents Pearson’s correlation coefficients between the SCR variable and other key variables, including BRCS, CRC, SCD-Q, and Age. Pearson’s correlation coefficient (r) quantifies the strength and direction of linear relationships between variables. For SCR, significant correlations are observed with BRCS (*r* = 0.419, *p* < 0.001) and CRC (*r* = 0.184, *p* < 0.01). These findings suggest that individuals with higher SCR scores, indicative of stronger modifiable lifestyle factors, tend to display greater resilience and cognitive reserve, supporting the hypotheses H2 and H3. This aligns with the convergent validity of SCR in relation to resilience and cognitive reserve. Furthermore, the significant association between SCR scores and indicators of resilience and cognitive health underpins its criterion validity in these areas. Additionally, there is no significant correlation between SCR and Age, supporting H4. When controlling for gender, results were consistent, except for a statistically significant relationship between BRCS and CRC (*r* = 0.143, *p* < 0.05).

**Table 4:** Pearson’s correlations (*N* = 402).

Variable	Mean (*SD*)	Age	BRCS	SCR	CRC	SCD-Q
Age	35.51 (14.94)	–				
BRCS	14.77 (2.79)	−0.028	–			
SCR	30.42 (4.53)	0.040	0.419 ***	–		
CRC	15.73 (3.27)	0.332 ***	0.110*	0.184**	–	
SCD-Q	5.79 (5.29)	0.051	−0.170 *	−0.381***	−0.123*	–

*p < 0.05, **p < 0.01, ***p < 0.001.

The Table 5 displays Pearson’s Correlations for each of the items of the proposed new SCR scale and the variables of interest in the study. Noticeably, none of the items correlated with the age variable. Age only exhibited an inverse correlation with CRC scores. On the other hand, the strongest correlation with resilience was observed with the item “willingness to learn new things.

**Table 5:** Pearson’s correlations across SCR items and variables of interest (*N* = 402).

		Spearman’s rho	
Age	BRCS	−0.028	
		CRC	0.332	***
		SCD-Q	0.051	
		Nutrition	0.115	*
		Physical condition	0.023	
		Sleeping	0.056	
		Cognition	0.077	
		Willingness to learn	0.008	
		Socialization	0.031	
		General health	−0.066	
		Vital plan	−0.060	
BRCS	CRC	0.110	*
	SCD-Q	−0.170	***
	Nutrition	0.213	***
	Physical condition	0.176	***
	Sleeping	0.167	***
	Cognition	0.253	***
	Willingness to learn	0.439	***
	Socialization	0.272	***
	General health	0.223	***
	Vital plan	0.424	***
CRC	SCD-Q	−0.123	*
	Nutrition	0.126	*
	Physical condition	0.114	*
	Sleeping	0.043	
	Cognition	0.201	***
	Willingness to learn	0.219	***
	Socialization	0.146	**
	General health	0.050	
	Vital plan	0.044	
SCD-Q	Nutrition	−0.229	***
	Physical condition	−0.160	**
	Sleeping	−0.148	**
	Cognition	−0.409	***
	Willingness to learn	−0.202	***
	Socialization	−0.287	***
	General health	−0.275	***
	Vital plan	−0.261	***
Nutrition	Physical condition	0.554	***
	Sleeping	0.245	***
	Cognition	0.314	***
	Willingness to learn	0.206	***
	Socialization	0.178	***
	General health	0.437	***
	Vital plan	0.286	***
Physical condition	Sleeping	0.198	***
	Cognition	0.219	***
	Willingness to learn	0.165	***
	Socialization	0.193	***
	General health	0.363	***
	Vital plan	0.249	***
Sleeping	Cognition	0.275	***
	Willingness to learn	0.159	**
	Socialization	0.173	***
	General health	0.265	***
	Vital plan	0.222	***
Cognition	Willingness to learn	0.382	***
	Socialization	0.342	***
	General health	0.279	***
	Vital plan	0.369	***
Willingness to learn	Socialization	0.348	***
	General health	0.282	***
	Vital plan	0.445	***
Socialization	General health	0.378	***
	Vital plan	0.400	***
General health	Vital plan	0.442	***

*p < 0.05, **p < 0.01, ***p < 0.001.

### Linear regression

The Linear Regression statistics support the adequacy of the alternative model in explaining the variability in the SCD-Q scores through BRCS, SCR, CRC, and Age. This model was statistically significant: *F*_(4, 401)_ = 18.40; MSE = 23.88; *p* < 0.001; *R*^2^ = 0.156. Table 6 depicts the linear regression coefficients.

**Table 6:** Linear regression coefficients (*N* = 402).

							95% CI	
Model		Unstandardized	Standard error	Standardized	*t*	*p*	Lower	Upper
H₀	(Intercept)	5.794	0.264		21.941	< 0.001	5.274	6.313
H₁	(Intercept)	20.028	1.996		10.036	< 0.001	16.105	23.951
	BRCS	−0.006	0.096	−0.003	−0.063	0.950	−0.196	0.184
	SCR	−0.430	0.060	−0.368	−7.161	< 0.001	−0.548	−0.312
	CRC	−0.140	0.080	−0.087	−1.740	0.083	−0.298	0.018
	Edad	0.034	0.017	0.095	1.938	0.053	−4.875 × 10^−4^	0.068

The standardized coefficients indicate the strength and direction of the relationship between each predictor variable and the outcome variable. However, only the SCR has a statistically significant standardized coefficient of −0.368, suggesting a negative relationship with the outcome variable. The collinearity statistics (tolerance and variance inflation factor, VIF) showed values closer to 1, indicating low multicollinearity.

## Discussion

By examining the SCR psychometric properties across various domains and its associations with BRCS scores to measure resilience and age, this study aims to provide a new tool in the field for the general population.

The current results indicate an adequate goodness of fit for the model, providing preliminary evidence of the construct validity of the SCR scale. More specifically, for the CFA, the *χ*^2^ test showed a significant improvement in fit for the factor model compared to the baseline model, supporting H1. Although the *χ*^2^ can be sensitive to sample size, additional fit indices—CFI, TLI, NNFI, and NFI—indicate a moderate to good fit. The RMSEA slightly exceeded the recommended threshold, suggesting potential for model refinement. Thus, the results support the first hypothesis regarding the SCR scale’s strong psychometric properties, encompassing variables such as nutrition, physical condition, sleeping patterns, cognition, willingness to learn, socialization, general health, and vital plan. This also supports the idea that individuals perceive their health and cognitive abilities (Lachman et al., 2011) endorsing the existing model of cognitive reserve. This also aligns with previous research highlighting the multidimensional nature of cognitive reserve (Jones et al., 2011; Stern, 2012).

The positive correlation between SCR scores and cognitive reserve questionnaires, as well as BRCS resilience measure, suggests that individuals with higher perceived cognitive reserve may also demonstrate greater resilience in coping with cognitive challenges. This finding supports hypothesis 2, indicating that individuals with heightened cognitive reserve are more likely to exhibit elevated levels of resilience. Moreover, it aligns with the concept that cognitive reserve reflects the brain’s capacity to withstand neuropathological damage and sustain cognitive function despite age-related changes (Cattaneo et al., 2022; McQuail et al., 2021). In this way, the identification of SCR as a strong predictor of self-perceived cognitive decline emphasizes the importance of cognitive reserve in maintaining cognitive function and subjective cognitive wellbeing across the lifespan. These findings underscore the potential utility of subjective assessments of cognitive reserve in identifying individuals at risk for cognitive decline and informing interventions aimed at preserving cognitive health.

Furthermore, the significant negative correlation between SCR scores and subjective cognitive decline scores supports hypothesis 3 and underscores the potential protective role of cognitive reserve against self-reported cognitive decline. This finding echoes previous research suggesting that higher levels of cognitive reserve may buffer against the subjective experience of cognitive decline, possibly by facilitating compensatory neural mechanisms or enhancing cognitive flexibility (Franzmeier et al., 2018; Pascual-Leone and Bartres-Faz, 2021).

The lack of relationship between SCR scores and chronological age supports hypothesis 4, highlighting the idea that subjective cognitive reserve remains independent of age. This finding is consistent with the concept that cognitive reserve is not solely determined by chronological age but is influenced by factors such as education, occupation, lifestyle, and cognitive engagement (Simon et al., 2023; Stern, 2002, 2012; Valenzuela and Sachdev, 2006). Nevertheless, the lack of correlation between SCR scores and subjective cognitive decline highlights the need for further investigation into the complex interplay between self-awareness of cognitive reserve and subjective perceptions of cognitive decline. Future research could explore potential moderators or mediators of this relationship to elucidate underlying mechanisms. It was surprising to find a correlation between CRC scores and age variables. It might be explained by a cohort effect, which is often observed in studies involving older populations. A cohort effect refers to differences that are attributed to the specific experiences or conditions that individuals from the same generation or age group have encountered. In the case of older populations, factors such as historical changes in education, lifestyle, or access to healthcare could influence cognitive reserve (CRC scores). These experiences might lead to a correlation between CRC scores and age, as older individuals may have developed distinct cognitive patterns based on the unique challenges and opportunities of their generation. Even though more research is needed in this area, if confirmed, this might indicate that SCR is less influenced by the level of education compared to other measures.

The study correlations reveal that scores in resilience, as measured by the BRCS, were positively correlated with all the items in the proposed SCR scale, encompassing domains such as Nutrition, Physical condition, Sleeping, Cognition, willingness to learn, socialization, general health, and vital plan. Interestingly, the variable “Willingness to learn” exhibited the strongest correlation with resilience scores. This suggests that individuals who display a greater propensity to learn new things may also exhibit higher levels of resilience. Previous research supports this idea, showing that lifelong learning is linked to better cognitive function and wellbeing, both of which boost resilience. Essentially, resilient individuals are often open to learning, adaptable, and proactive in overcoming challenges, which fits with the cognitive reserve framework (Leipold and Greve, 2009; Moret-Tatay et al., 2015; Murphy et al., 2021).

There are several limitations that should be addressed. Firstly, the adoption of a cross-sectional design limits the ability to establish causal relationships, emphasizing the need for longitudinal studies to clarify the temporal dynamics of SCR and its impact on cognitive outcomes and resilience. Additionally, the sample size for the test–retest reliability was relatively small, which may affect the stability of these findings. Moreover, it should be noted that participants were asked to report any health issues, but this information was not verified, so we acknowledge that not controlling for potential confounding variables, such as comorbidities, medication use, and socioeconomic status, is a limitation. Future research should account for these factors to better isolate the unique contribution of SCR to cognitive wellbeing. Furthermore, the reliance on self-report measures for SCR, resilience, and subjective cognitive decline may introduce biases, suggesting the need to integrate objective measures of cognitive functioning for a more comprehensive understanding. While SCR demonstrated short-term predictive validity for self-perceived cognitive decline, its long-term predictive capacity remains uncertain, warranting longitudinal studies to assess its ability to predict actual cognitive decline and dementia risk over extended periods. In this regard, replication with groups affected by cognitive impairment would be valuable.

Overall, this research offers significant contributions to understanding how subjective cognitive reserve can be assessed in the broader population and its relevance for cognitive aging, resilience, and subjective cognitive decline. Continued investigation in this field holds promise for informing strategies to enhance cognitive health and overall wellbeing throughout one’s lifetime.

## Conclusion

The study aimed to examine validate a new scale measuring subjective cognitive reserve (SCR) across multiple domains and examine its relationship with resilience and age. Results showed robust psychometric properties for the SCR scale, supporting the existing model of perceived cognitive reserve. Positive correlations were found between SCR scores and measures of cognitive reserve (CRC) and resilience (BRCS), indicating consistency across assessment tools and suggesting that individuals with higher cognitive reserve may also have greater resilience. Additionally, a significant negative correlation was observed between SCR scores and self-reported cognitive decline (SCD-Q), suggesting an inverse relationship between awareness of cognitive reserve and subjective decline. Notably, no relationship was found between SCR scores and chronological age, underscoring the independence of SCR from age and highlighting the importance of cognitive reserve regardless of age.

## Data Availability

The raw data supporting the conclusions of this article will be made available by the authors, without undue reservation.

## Appendix A

### SCR items

Indique de 1 (nada satisfecho) a 5 (muy satisfecho) el grado de satisfacción con las siguientes áreas de su vida

1. La calidad de su nutrición
2. Su condición física
3. Su higiene de sueño
4. Su capacidad cognitiva general
5. Sus ganas de aprender cosas nuevas
6. Su socialización y apoyo social
7. Su salud general
8. Sus objetivos y plan de vida

Translation: Rate from 1 (very dissatisfied) to 5 (very satisfied) the level of satisfaction with the following areas of your life:

1. The quality of your nutrition.
2. Your physical condition.
3. Your sleep hygiene.
4. Your overall cognitive ability.
5. Your willingness to learn new things.
6. Your socialization and social support.
7. Your overall health.
8. Your goals and Vital plan.

*Labels: 1 = Nutrition, 2 = Physical condition, 3 = Sleeping, 4 = Cognition, 5 = Willingness to learn, 6 = Socialization, 7 = General health, and 8 = Vital plan.
